# Performance of intraoperative artificial intelligence-assisted label-free stimulated Raman histology in recurrent brain tumors

**DOI:** 10.1093/noajnl/vdag267

**Published:** 2026-10-29

**Authors:** Gina Fürtjes, Abdulkader Al Shughri, Thomas Stehle, David Reinecke, Anna-Katharina Meißner, Nina Jung, Zeynep Mercan, Nicolas Neidert, Pierre Scheffler, Lili Leyer, Claire Wang, Zachari Gueorguiev, Niklas von Spreckelsen, Daniel Ruess, Christian Freudiger, Florian Khalid, Adrian Ion-Margineanu, Roland Goldbrunner, Jürgen Beck, Volker Neuschmelting, Jakob Straehle

**Affiliations:** Department of General Neurosurgery, Centre of Neurosurgery, University Hospital Cologne, Cologne, Germany; Institute for Neuropathology, University Hospital Cologne, University of Cologne, Cologne, Germany; Institute for Neuropathology, University Hospital Cologne, University of Cologne, Cologne, Germany; Department of General Neurosurgery, Centre of Neurosurgery, University Hospital Cologne, Cologne, Germany; Department of General Neurosurgery, Centre of Neurosurgery, University Hospital Cologne, Cologne, Germany; Department of General Neurosurgery, Centre of Neurosurgery, University Hospital Cologne, Cologne, Germany; Department of Neurosurgery, Medical Center, University of Freiburg, Freiburg, Germany; Center for Advanced Surgical Tissue Analysis (CAST), Medical Center, University of Freiburg, Freiburg, Germany; Department of Neurosurgery, Medical Center, University of Freiburg, Freiburg, Germany; Center for Advanced Surgical Tissue Analysis (CAST), Medical Center, University of Freiburg, Freiburg, Germany; Department of Neurosurgery, Medical Center, University of Freiburg, Freiburg, Germany; Center for Advanced Surgical Tissue Analysis (CAST), Medical Center, University of Freiburg, Freiburg, Germany; Department of General Neurosurgery, Centre of Neurosurgery, University Hospital Cologne, Cologne, Germany; Department of General Neurosurgery, Centre of Neurosurgery, University Hospital Cologne, Cologne, Germany; Department of General Neurosurgery, Centre of Neurosurgery, University Hospital Cologne, Cologne, Germany; Department of Neurosurgery, West Coast Clinic Heide, Heide, Germany; Department of Stereotaxy and Functional Neurosurgery, Centre of Neurosurgery, University Hospital Cologne, Cologne, Germany; Invenio Imaging Inc, Santa Clara, California, USA; Invenio Imaging Inc, Santa Clara, California, USA; Invenio Imaging Inc, Santa Clara, California, USA; Department of General Neurosurgery, Centre of Neurosurgery, University Hospital Cologne, Cologne, Germany; Department of Neurosurgery, Medical Center, University of Freiburg, Freiburg, Germany; Center for Advanced Surgical Tissue Analysis (CAST), Medical Center, University of Freiburg, Freiburg, Germany; Department of General Neurosurgery, Centre of Neurosurgery, University Hospital Cologne, Cologne, Germany; Department of Neurosurgery, Medical Center, University of Freiburg, Freiburg, Germany; Center for Advanced Surgical Tissue Analysis (CAST), Medical Center, University of Freiburg, Freiburg, Germany

**Keywords:** artificial intelligence, intraoperative diagnosis, radiation necrosis, recurrent brain tumors, stimulated Raman histology

## Abstract

**Background:**

Therapy-induced tissue alterations complicate intraoperative diagnosis of recurrent central nervous system (CNS) tumors. We evaluated stimulated Raman histology (SRH) combined with convolutional neural networks (CNNs) for rapid tumor detection and classification.

**Methods:**

In this bicentric observational study, fresh tissue from patients undergoing resection or stereotactic biopsy for suspected recurrent CNS tumors was imaged with fiber-laser-based SRH. CNN1 performed binary tumor/non-tumor prediction and CNN2 histotype classification. CNN1 was evaluated against blinded neuropathologist SRH consensus and CNN2 against the final integrated histopathological diagnosis.

**Results:**

Of 382 acquired SRH images, 262 images from 143 patients were included. CNN1 achieved an AUC of 0.975 (95% CI, 0.95-1.00) for tumor detection. An exploratory cohort-derived tumor probability threshold of ≥59 increased specificity from 70.7% to 92.0% while maintaining 95.2% sensitivity and identifying 71.4% of radiation necrosis samples. Among 212 images in treatment subgroup analysis, CNN1 accuracy did not differ significantly across prior-treatment groups (overall 90.1%; *P* = .080). CNN2 accuracy declined from 61.8% after surgery alone to 39.0% after surgery, radiotherapy, and medical treatment (χ^2^ = 6.04, *P* = .049). Multimodal therapy was independently associated with lower odds of correct CNN2 prediction (adjusted OR, 0.10 [95% CI, 0.02-0.39]; *P* = .001 and 0.04 [95% CI, 0.01-0.20]; *P* < .001). 120/382 images (31.4%) were of insufficient diagnostic quality; application of the quality control threshold would have rejected 84/120 (70.0%).

**Conclusions:**

CNN1 maintained high tumor/non-tumor discrimination across treatment subgroups when image quality was sufficient, whereas CNN2 entity-classification performance declined with increasing treatment intensity. Reliable SRH-based diagnosis in recurrent tumors requires validated image-quality control measures and model adaptation to recurrent disease.

Key Points

- CNN1 threshold ≥59 improved specificity while maintaining high sensitivity.
- CNN2 entity-classification accuracy declined with increasing treatment intensity.
- Strict image-quality control is essential for reliable SRH diagnosis.

Importance of the Study

Stimulated Raman histology enables rapid, label-free intraoperative tissue assessment, but current AI models have been developed predominantly in treatment-naïve tumors. This bicentric real-world study evaluates their performance specifically in suspected recurrent central nervous system (CNS) tumors, where treatment-related changes and radiation necrosis complicate diagnosis. Binary tumor recognition remained robust when image quality was sufficient, whereas CNN2 entity classification deteriorated with increasing treatment intensity. Nearly one-third of acquired images were diagnostically insufficient, and automated quality screening would have rejected 70% of these. The findings define both the strengths and current limitations of SRH-based AI in recurrent disease and support prospective validation of image-quality control, independent validation of recurrence-specific diagnostic thresholds, and development of models tailored to treatment-altered tissue.

Recurrent brain tumors represent a frequent challenge in neuro-oncological practice, particularly as improved oncological treatments have prolonged progression-free and overall survival in many tumor entities. Local recurrence remains common across brain metastases,^1-4^ meningiomas,^5^ and glioblastoma,^6^ although rates vary substantially according to tumor type, grade, treatment, and extent of resection.

Reliable noninvasive differentiation of viable recurrent tumor from treatment-related tissue alterations remains difficult. Radiation necrosis may closely mimic tumor recurrence on conventional MRI,^7^ while diffusion-weighted imaging, perfusion MRI, and MR spectroscopy improve characterization but retain imperfect diagnostic accuracy.^8-10^ Amino-acid PET, particularly ^1^^8^F-FET PET, can further improve discrimination between recurrence and treatment-related injury^11^ but does not eliminate diagnostic uncertainty.^12^

Radiation necrosis is also clinically relevant in increasingly long-term survivors after radiotherapy and may occur across different irradiated brain tumor entities. Its occurrence is influenced by radiation dose and fractionation, concomitant systemic therapy, and patient-related factors.^13-15^

This diagnostic challenge extends to the intraoperative setting. Surgical strategy and extent of resection may depend on rapid assessment of whether sampled tissue represents a viable tumor or a predominantly non-neoplastic treatment effect. Conventional intraoperative frozen-section histology remains the current standard but is affected by sampling error, tissue-processing artifacts, and interpretative uncertainty.^16^ These limitations may be particularly relevant in recurrent disease, where viable tumor can coexist with fibrosis, microcalcification, neovascularization, necrosis, and reactive cellular alterations.^17^ Frozen-section concordance with the final formalin-fixed paraffin-embedded diagnosis is high overall but lower in re-do surgery and selected entities, underscoring the difficulty of treatment-altered specimens.^18^^,^^19^

Stimulated Raman histology (SRH) enables rapid, label-free microscopic imaging of fresh surgical tissue without fixation, sectioning, or staining. In combination with convolutional neural networks (CNNs), SRH has demonstrated high diagnostic performance for intraoperative tumor detection and classification within a few minutes. However, existing SRH-based AI models have been developed predominantly using treatment-naïve tumor tissue, while recurrent and treatment-altered specimens remain underrepresented in available training datasets. Their performance in the morphologically heterogeneous setting of recurrent disease, particularly after multimodal treatment and in the presence of radiation necrosis, therefore, remains insufficiently characterized.^20-24^

We conducted a prospective bicentric real-world study of patients undergoing resection or stereotactic biopsy for suspected recurrent CNS tumors. We evaluated a binary tumor/non-tumor CNN and a tumor-entity classification CNN, investigated the association between prior treatment intensity and diagnostic performance, and assessed automated image-quality metrics as a prerequisite for reliable AI-assisted SRH interpretation in recurrent disease.

## Methods

In a prospective bicentric observational real-world study design, patients with suspected local recurrent brain tumor disease were recruited in 07/2021-03/2025 after an interdisciplinary neuro-oncological tumor board indicated resection or stereotactic biopsy. The study protocol has been approved by the local ethics committees (Cologne: 21-1238; Freiburg: 23-1174-S1, 23-1175-S1). All patients gave their written informed consent for the scientific use of their data according to the European law.

The following inclusion criteria were defined: (1) tumor-suspect lesions of the central nervous system, (2) age over 18 years, and (3) written informed consent provided either by the patient or, in cases of impaired decisional capacity, by a legally authorized representative in accordance with the local ethics approval. The following exclusion criteria were set: (1) absence of written informed consent by the patient or a legally authorized representative, or (2) withdrawal of consent. If the collected specimen was inadequate, eg broken slide or specimen sample size below 1.7 mm in diameter for SRH imaging or SRH images evaluated as low quality by 2 independent neuropathologists, these specimen samples were excluded from analysis.

### Specimen Collection and Intraoperative SRH Image Acquisition

Small tissue samples (1-3 mm^3^) were obtained intraoperatively by the operating neurosurgeon using a microtumor forceps (B. Braun SE, Melsungen, Germany). Tissue was selected from an area considered macroscopically representative of the suspected recurrent tumor, preferentially from the tumor core. The specimen was placed on a plastic slide, gently compressed with a cover glass, and imaged using a fiber-laser-based stimulated Raman spectroscopy microscope (NIO, Invenio Imaging Inc., Santa Clara, CA, USA). The system comprises four main modules^25^: (1) a fiber-coupled microscope; (2) a dual-wavelength fiber-laser source (790 and 1020 nm); (3) a combined laser/microscope control unit; and (4) a computer for image processing, display, and CNN analysis. Tissue contrast in the images arises from differences in Raman scattering spectra due to varying protein and lipid composition. Image acquisition was performed at 2 Raman shifts—2845 cm^−1^ (CH_2_- stretching mode primarily for lipids) and 2940 cm^−1^ (CH_3_- stretching mode primarily for protein and nucleic acids). These signals were combined to generate a virtual H&E-like SRH image using proprietary software (Invenio Imaging). A mosaic of 400 × 400 μm fields of view were captured at 2 s per frame, and subsequent image processing generated a virtual H&E-like image covering an area of 3.06 mm^2^ within 3 minutes.^2^^,^^26^

### Neuropathological Review

All SRH images were independently reviewed by 2 board-certified neuropathologists with specific training in SRH interpretation. Each image was classified into 1 of the following predefined categories: tumor, non-tumor, radiation necrosis, or low-quality. The reviewers were blinded to the intraoperative diagnosis, frozen section results, and final histopathological reports at the time of evaluation. In cases of disagreement, a consensus diagnosis was established in a joint review session. For image-level analyses of tumor presence and image quality, this consensus SRH classification served as the reference standard. For entity-level CNN2 analyses, when tumor tissue was confirmed, the final integrated FFPE-based histopathological diagnosis of the corresponding case served as the reference standard. Of note, SRH images classified as low quality by neuropathologist consensus were excluded from diagnostic performance analyses because no reliable image-level ground-truth diagnosis could be assigned.

### Application of AI-Based Analysis Tools

Two independent CNN-based tools were used for brain tumor diagnostics as previously reported.^20^^,^^21^ CNN1 performs binary tumor/non-tumor discrimination, whereas CNN2 performs multiclass histotype classification; CNN2 output classes can additionally be collapsed post hoc into tumorous and non-tumorous categories.

CNN1 (GliomaReveal, Invenio Imaging Inc., Santa Clara, California, USA) is based on the model previously reported by Reinecke et al.^21^ It discriminates between (1) tumor and (2) non-tumor using a tumor probability threshold of 40 (tumor ≥40; non-tumor <40), after an automated check confirming at least 39 high-quality patches (300 × 300 pixels) per SRH image. In brief, the CNN was trained on an annotated dataset of 570 whole-slide SRH images, including all primary and recurrent neurooncological tumor entities. Training was terminated when preliminary validation accuracy exceeded 95% and loss was below 0.10. The CNN had been retrained a second and a third time with different random seeds on the same external dataset for reliability and ROC analysis. In tissue samples valued as diagnostic a tumor probability score ≥40 had been proposed to be suspicious for tumor tissue, while a probability score <40 had been set as cutoff value for non-tumorous tissue.

In parallel, CNN1-derived patch-level quality information was used for automated image quality assessment. Each patch was processed by the CNN and classified as low quality when the highest predicted class probability corresponded to the low-quality class; no additional manually defined threshold was applied. Low-quality patches were excluded from downstream diagnostic classification, which was based on the remaining high-quality patches. During the original analysis workflow, diagnostic CNN inference required at least 39 high-quality patches per SRH image. No additional image-level exclusion criterion based on the proportion of low-quality patches was applied during the original CNN inference. Of note, the percentage-based image-quality threshold was based on the results of the present study and was not used to gate the original diagnostic CNN output.

CNN2 was previously reported by Hollon et al^20^ to classify the ten most common CNS tumor entities and 4 non-tumorous CNS tissue diagnoses, 14 classes in total: (1) ependymoma, (2) malignant glioma, (3) diffuse low-grade glioma, (4) pilocytic astrocytoma, (5) schwannoma, (6) medulloblastoma, (7) meningioma, (8) metastatic tumor, (9) pituitary adenoma, and (10) lymphoma as tumorous classes, as well as (11) nondiagnostic tissue, (12) gliosis, (13) grey matter, and (14) white matter as non-tumorous classes. CNN2 learned a hierarchy of recognizable histomorphological feature representations to classify the major histopathologic classes of brain tumors from a dataset including 415 patients with primary and recurrent disease using a categorical cross-entropy loss function weighted by inverse class frequency. A validation set was used for hyperparameter tuning and model selection based on patch-level classification accuracy. The entity class with the highest probability score was defined as the final CNN prediction. Both CNN models require approximately one minute for inference per case, indicating suitability for near real-time intraoperative application.

### Statistical Analysis

Statistical analysis was performed using IBM SPSS Statistics (Version 30, IBM Corp., Armonk, NY, USA). Continuous variables were summarized as means with standard deviations or medians with interquartile ranges, as appropriate.

To assess the image quality for each image, the percentage of low-quality patches was calculated from the number of CNN1-detected low-quality patches divided by the total number of analyzed patches. This metric was compared between neuropathologist-defined quality groups using the Mann-Whitney U test and summarized as median and interquartile range. Receiver operating characteristic (ROC) analysis was performed to evaluate its ability to predict neuropathologist-assessed low image quality. The optimal cutoff was determined using the Youden index, and sensitivity, specificity, accuracy, positive predictive value, and negative predictive value were calculated. To simulate the potential effect of an automated image-level quality gate, the Youden-derived threshold was post-hoc applied to all acquired SRH images. Images exceeding the cutoff value were considered quality-control failures, whereas images at or below the cutoff were considered eligible for downstream diagnostic inference. This post-hoc workflow simulation was compared with the neuropathologist consensus quality assessment and was not part of the original CNN inference procedure.

Diagnostic performance of both CNNs was evaluated using confusion matrices with calculation of sensitivity, specificity, positive and negative predictive values, accuracy, and *F*1-scores. For binary tasks, ROC curves and areas under the curve (AUCs) with 95% confidence intervals were derived. For CNN2, the maximum prediction score per image, reflecting the model’s most confident class assignment, was used to assess confidence in correct histotype classification. ROC analysis of these maximal scores assessed their ability to distinguish correct from incorrect histotype predictions. Separately, CNN2 output classes were collapsed post hoc into tumor and non-tumor categories to evaluate broad tumor recognition. Values >0.8 were considered excellent and >0.9 outstanding. For exploratory threshold optimization, the Youden index was applied to the respective model output scores on the full cohort. Misclassification patterns were further explored, with particular focus on radiation necrosis as a clinically relevant differential diagnosis in recurrent brain malignancies.

To assess potential effects of prior treatment on diagnostic accuracy, a binary variable (correct vs. incorrect CNN prediction) was tested against previous treatment categories (surgery; surgery and radiotherapy; surgery, radiotherapy, and medical treatment) using chi-square and multivariable logistic regression models adjusted for patient age and histopathological category. Final histopathological diagnoses were consolidated into four main pathology groups to ensure model stability and avoid sparse-cell bias: Metastases, meningioma, malignant glioma, and others (all remaining entities). These were included as categorical covariates in logistic regression. Separate models were computed for both CNNs. Two-sided *P*-values <.05 were considered statistically significant.

## Results

### Image Quality Assessment

Robust image-quality control is essential for reliable AI-assisted SRH diagnostics, particularly in recurrent tumors with therapy-associated tissue alterations. In total, 382 SRH images were independently assessed by 2 neuropathologists, of which 262 (68.6%) were considered of sufficient diagnostic quality and 120 (31.4%) were classified as low quality. Neuropathologist-rated low-quality SRH images showed a substantially higher proportion of CNN1-derived low-quality patches than images rated as diagnostically sufficient (Figure 1A). The median proportion of low-quality patches was 32.7% (IQR 16.5-54.1) in images classified as low quality versus 8.9% (IQR 3.5-20.5) in images classified as sufficient quality. Mean values showed a similar pattern, with 37.7 ± 26.8% low-quality patches in the low-quality group compared with 15.0 ± 16.6% in the sufficient-quality group. This difference was statistically significant (Mann-Whitney *U* = 6910.5, *P* < .001).

**Figure 1.**
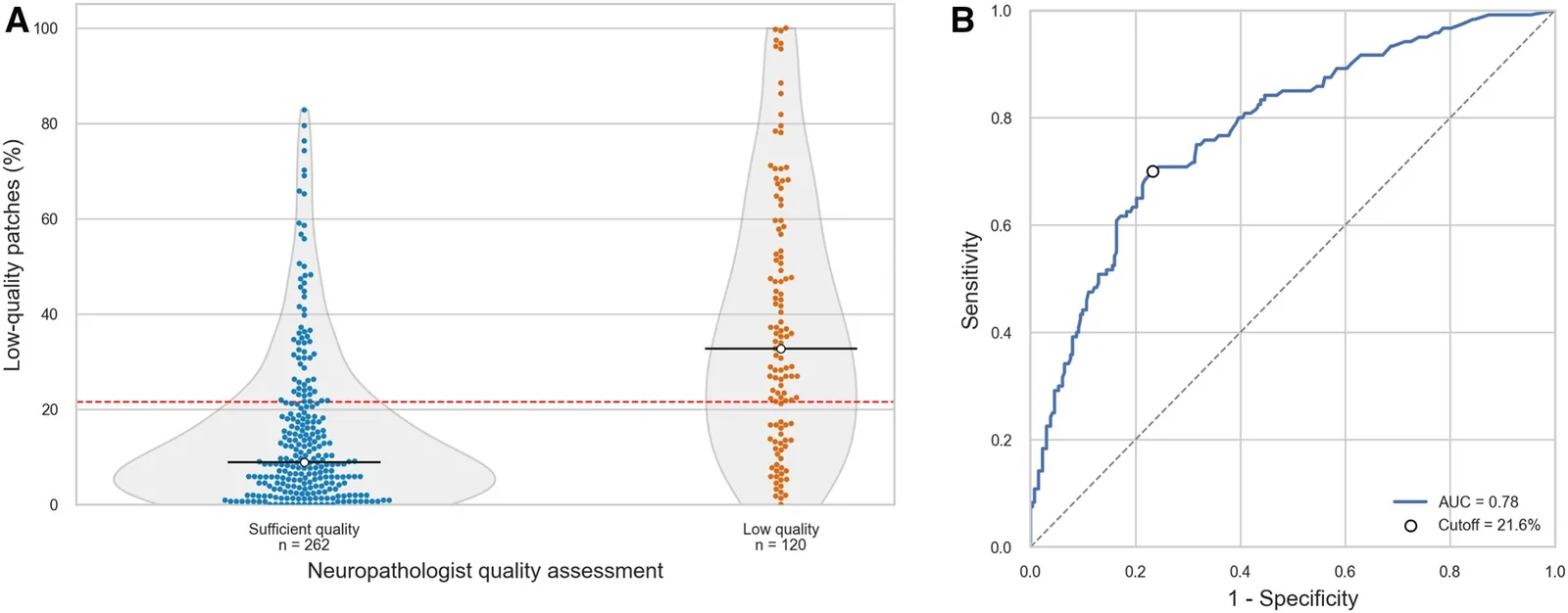
CNN1-derived image quality assessment compared with neuropathologist-based quality rating. (A) AI-derived low-quality patch proportion. Distribution of CNN1-derived low-quality patch proportions in SRH images rated as either sufficient quality or low quality by the neuropathologist. Each dot represents one SRH image. Violin plots show the distribution, with horizontal bars and circles indicating the median. The dashed line marks the Youden-optimized cutoff of 21.6% low-quality patches. (B) Receiver operating characteristic (ROC) analysis for low-quality image prediction. Receiver operating characteristic curve assessing the ability of the AI-derived low-quality patch proportion to predict neuropathologist-assessed low image quality. The analysis yielded an AUC of 0.78.

ROC analysis demonstrated good, but incomplete, discrimination of neuropathologist-assessed low image quality by the AI-derived low-quality-patch proportion, with an AUC of 0.78 (Figure 1B). The Youden-derived threshold of 21.6% low-quality patches yielded a sensitivity of 70.0%, specificity of 76.7%, and overall accuracy of 74.6%, with a positive predictive value of 57.9% and a negative predictive value of 84.8%.

Importantly, the existing minimum-patch prerequisite did not provide sufficient image-level quality control in the recurrent tumor samples included in this study. Of the 120 images classified as diagnostically insufficient by neuropathologist consensus, 110 (91.7%) nevertheless contained at least 39 high-quality patches and were therefore eligible for diagnostic CNN inference under the original workflow. In a simulation of the 21.6% low-quality-patch threshold as an image-level quality-control gate, 84/120 (70.0%) neuropathologist-defined low-quality images would have been rejected, whereas 36/120 (30.0%) would have passed the automated quality criterion. Among the 262 diagnostically sufficient images, 201/262 (76.7%) would have passed the quality gate, and 61/262 (23.3%) would have been rejected. Thus, 237/382 images would have been eligible for downstream diagnostic inference, of which 36/237 (15.2%) would nevertheless have been considered non-diagnostic by neuropathologist consensus.

### Demographic and Baseline Characteristics

A total of 262 SRH images deriving from 143 patients with suspected CNS tumor recurrency were included in this study. Of these, 173 SRH images were obtained from 87 patients at the University Hospital Cologne and 89 images from 56 patients at the University Hospital Freiburg. The cohort comprised 68 female (47.6%) and 75 male (52.4%) patients with a mean age of 57.7 years (range 21-88 years, SD ±15.2).

Final histopathological results show tumor tissue in 74.8% of patients, and diagnoses were distributed as follows: malignant gliomas (30.8%), meningiomas (20.3%), metastases (16.1%), low-grade gliomas (3.5%), pituitary neuroendocrine tumors (2.8%), and ependymomas (1.4%). In 25.2% of patients, the specimen did not contain tumor tissue gliosis (18.9%), radiation necrosis (6.3%). Detailed subgroup information and distribution of SRH images are provided in Table 1.

**Table 1.** Distribution of histopathological entities across patients and corresponding SRH images

Entity	Patients, *n*	Patients, %	SRH images, *n*	SRH images, %
Recurrent tumors				
Malignant glioma				
Oligodendroglioma CNS WHO 3	5	3.50	8	3.05
Astrocytoma CNS WHO 3	4	2.80	5	1.91
Astrocytoma CNS WHO 4	3	2.10	3	1.15
Glioblastoma CNS WHO 4	27	18.88	49	18.70
Gliosarcoma CNS WHO 4	1	0.70	1	0.38
Pleomorphic xanthoastrocytoma CNS WHO 3	1	0.70	1	0.38
High-grade astrocytoma with piloid features	1	0.70	1	0.38
Astroblastoma	1	0.70	2	0.76
Pediatric-type diffuse high-grade glioma	1	0.70	1	0.38
Low-grade glioma				
Oligodendroglioma CNS WHO 2	3	2.10	6	2.29
Astrocytoma CNS WHO 2	2	1.40	2	0.76
Metastases				
NSCLC	11	7.69	22	8.40
SCLC	4	2.80	9	3.44
Melanoma	2	1.40	5	1.91
Mammary carcinoma	2	1.40	2	0.76
Adenocarcinoma of the gastrointestinal tract	2	1.40	5	1.91
Prostate carcinoma	1	0.70	1	0.38
Large-cell NET	1	0.70	3	1.15
Meningioma				
CNS WHO 1	7	4.90	12	4.58
CNS WHO 2	16	11.19	29	11.07
CNS WHO 3	6	4.20	12	4.58
Other glial tumors				
Ependymoma CNS WHO 3	2	1.40	2	0.76
Pituitary tumors				
Pituitary neuroendocrine tumors	4	2.80	6	2.29
No tumor				
Radiation necrosis	9	6.29	14	5.34
Gliosis/no tumor	27	18.88	61	23.28
Total	**143**	**100.00**	**262**	**100.00**

Among all patients, 21.7% received unimodal and 78.3% multimodal therapy prior to inclusion in this study. Unimodal treatments predominantly consisted of surgery (15.4%), stereotactic radiosurgery (SRS, 2.1%), and brachytherapy (2.1%), whereas fractionated photon radiotherapy (fRT, 0.7%) and medical treatment (chemotherapy/immunotherapy) alone (1.4%) were infrequent. In the multimodal cohort, the most common regimen prior to this study was a trimodal combination of radiotherapy, surgery, and chemotherapy (in total 37.8%; fRT 37.1% and SRS 0.7%), less frequent combinations of radiotherapy and surgery (total: 20.3%; fRT: 15.4%; SRS: 4.2%; BT: 0.7%), and a quadruple regimen including immunotherapy in addition (4.2%). Further details see also Figure 2A.

**Figure 2.**
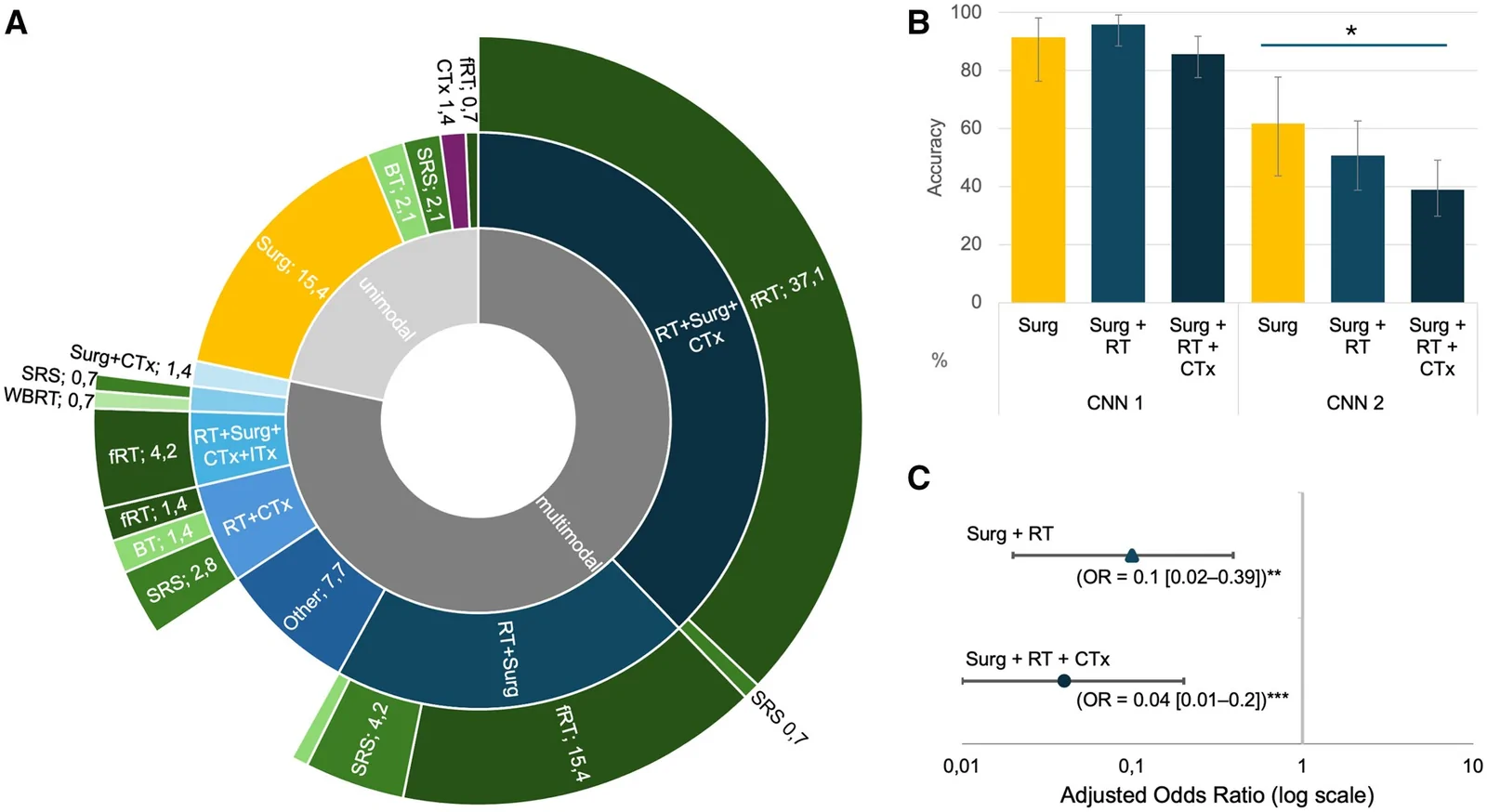
Prior treatment modalities and the effect on prediction models. (A) Distribution of previous treatment approaches among all patients. The inner ring differentiates between unimodal (21.7%) and multimodal therapy (78.3%), while the outer rings specify corresponding treatment modalities and subtypes. Unimodal therapy mainly consisted of surgery (15.4%), whereas multimodal regimens were dominated by the trimodal combination of radiotherapy, surgery, and chemotherapy (37.8%), followed by dual combinations such as radiotherapy and surgery (20.3%) and radiotherapy and medical treatment (5.6%). The third ring specifies the different kinds of radiotherapy per multimodal treatment group. (B) Accuracy of CNN 1 and 2 depending on prior therapy. Bar plot showing unadjusted classification accuracy (%) of CNN 1 and CNN 2 across previous treatment groups (surgery, surgery + radiotherapy, surgery + radiotherapy + medical treatment). Error bars indicate 95% confidence intervals for binomial proportions. For CNN 1, accuracy remained uniformly high across all therapy groups (91.2%, 95.9%, and 85.7%, respectively; χ^2^ = 5.05, *P* = .080). In contrast, CNN 2 showed a stepwise decline in accuracy with increasing multimodality (61.8%, 50.7%, and 39.0%, respectively; χ^2^ = 6.04, *P* = .049; linear trend *P* = .014), suggesting that prior combined treatment—particularly trimodal therapy—was associated with reduced diagnostic performance. (C) Adjusted logistic regression of CNN2 classification accuracy by treatment history. Forest plot displaying adjusted odds ratios (log scale) for correct CNN2 prediction across prior treatment groups. Patients with surgery only served as the reference category (OR = 1, vertical line). Odds ratios were derived from multivariable logistic regression models including age and pathology as covariates. Both multimodal therapy groups showed substantially reduced odds of correct classification compared to surgery-only cases (Surgery + RT: adjusted OR = 0.10 [95% CI, 0.02-0.39]; Surgery + RT + CTx: adjusted OR = 0.04 [95% CI, 0.01-0.20]; *P* ≤ .001). * = *P* < .05; ** = *P* < .01; *** = *P* < .001. RT = radiotherapy; fRT = fractionated radiotherapy; SRS = stereotactic radiosurgery; BT = brachytherapy; WBRT = whole-brain radiotherapy; Surg = surgery; CTx = chemotherapy; ITx = immunotherapy.

### Validation of CNNs

When CNN1 (tumor/non-tumor prediction model) was applied as its intended use in glioma surgery, based on a tumor probability score of 40 to differentiate tumor from non-tumor, 185/187 tumor samples were correctly classified as tumor (sensitivity 98.9%), whereas 53/75 non-tumor samples (specificity 70.7%) were correctly identified. This corresponded to a positive predictive value (PPV) of 89.4% and a negative predictive value (NPV) of 96.4%. The *F*1-score was 93.9%, and overall accuracy was 90.8%. Of note, nearly one-third (5/14, 35.7%) SRH images of radiation necrosis were misclassified as tumor. Still, the ROC analysis yielded an outstanding AUC of 0.975 (95% CI, 0.95-1.0) (Figure 3A).

**Figure 3.**
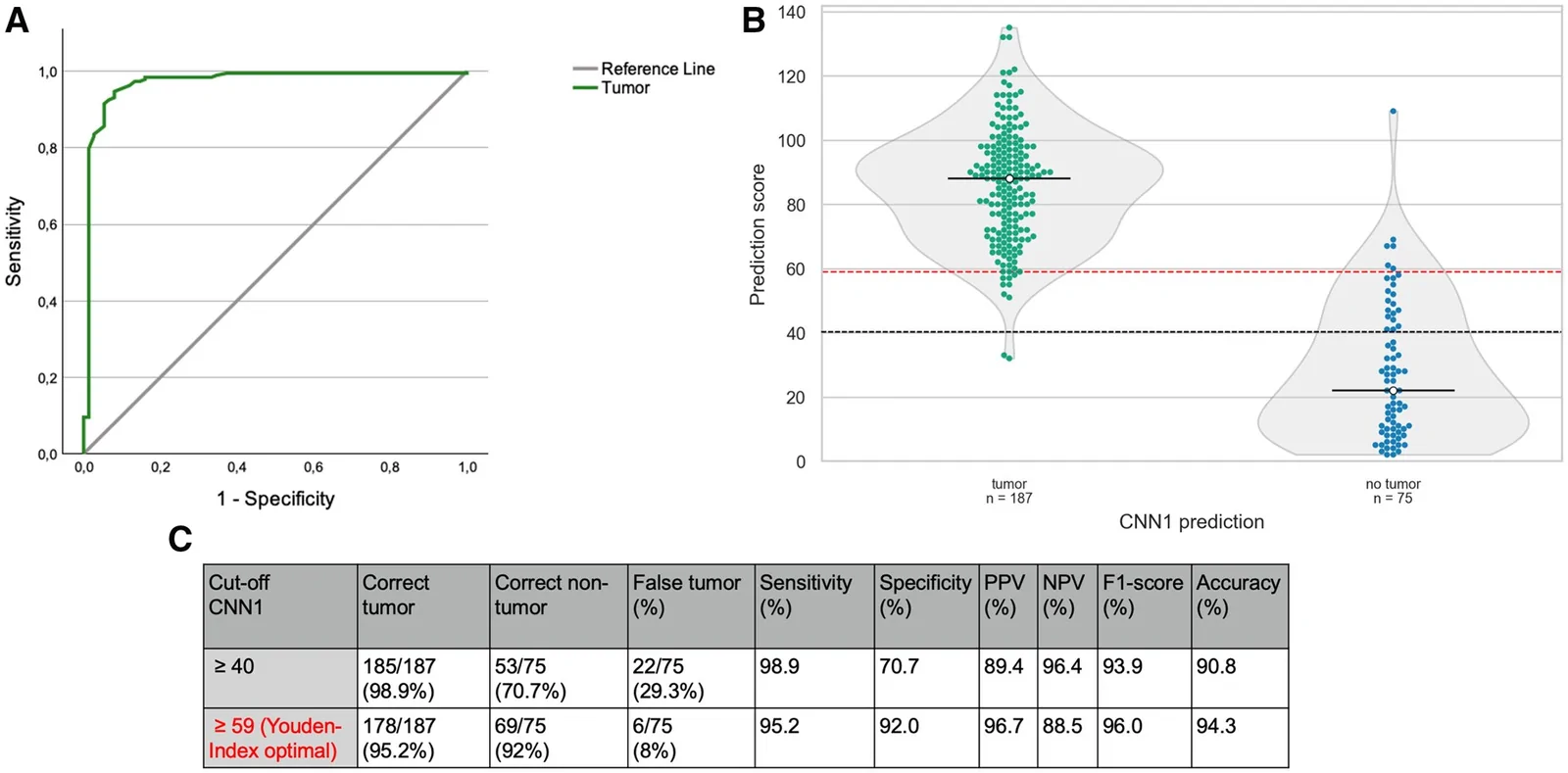
Performance metrics of CNN1 for tumor vs. non-tumor discrimination. (A) Receiver operating characteristic (ROC) analysis. ROC curve for CNN1 showing excellent overall discrimination between tumor and non-tumor samples (AUC = 0.975; 95% CI, 0.95-1.00). (B) Tumor probability score distribution. Violin/beeswarm plot illustrating tumor probability score distributions for tumor (*n* = 187) and non-tumor (*n* = 75) specimens. The dashed upper line indicates the exploratory cohort-derived threshold of ≥59 determined by the Youden index; the lower dashed line indicates the conventional threshold of 40 used in glioma surgery. Horizontal bars represent the median. (C) Performance comparison across applied thresholds. Performance summary for 2 relevant tumor probability score thresholds of CNN1. The optimized threshold determined by the Youden index (≥59) improved specificity to 92.0% while maintaining high sensitivity (95.2%), resulting in an overall accuracy of 94.3% and *F*1-score 96.0%.

In an exploratory analysis, the Youden index identified a tumor probability score threshold of 58.5 for differentiating tumorous from non-tumorous lesions in this recurrent tumor cohort (Figure 3B). When applied within the present cohort, this threshold maintained a high sensitivity of 95.2% while increasing specificity to 92.0%, with only 6 non-tumor images (8.0%) misclassified as tumor. The PPV resulted to 96.7%, NPV to 88.5%, *F*1-score of 96%, and overall accuracy could be improved to 94.3% (Figure 3C). Overall, the rate of non-tumor samples misclassified as tumor decreased substantially—from 22 of 75 cases (29.3%) before optimization to 6 of 75 cases (8%) after applying the specific threshold most suitable for CNS recurrencies. Importantly, 10 out of 14 radiation necrosis samples (71.4%) were correctly identified as non-tumor.

When CNN2 output classes collapsed into a binary tumor/non-tumor classification, 178/187 tumor samples (95.2%) were assigned to one of the tumorous classes and 57/75 non-tumor samples (76.0%) to a non-tumorous class, corresponding to an overall accuracy of 89.7%. Thus, CNN2 largely retained the ability to distinguish tumorous from non-tumorous tissue in the recurrent tumor setting. In contrast, performance was substantially lower for the intended task of correct histotype classification. Overall, 123/262 images (46.9%) were assigned to the correct diagnostic entity. Correctly classified samples showed higher maximal probability scores (median 83 vs. 54) (Figure 4B), with a significant shift toward higher values confirmed by a Mann-Whitney U test (mean rank 158.65 vs. 107.47; *U* = 5209, *P* < .001). ROC analysis assessing the ability of the maximal prediction score to distinguish correct from incorrect histotype predictions yielded an AUC of 0.73 (95% CI, 0.67-0.79) (Figure 4A). At the Youden-index-derived threshold of ≥64, sensitivity for identifying correct predictions was 62.6%, specificity 69.1%, and overall accuracy 66.0% (*F*1-score = 63.4%) (Figure 4C). These findings indicate that the principal limitation of CNN2 in recurrent tumors concerned accurate entity classification rather than broad recognition of tumor tissue.

**Figure 4.**
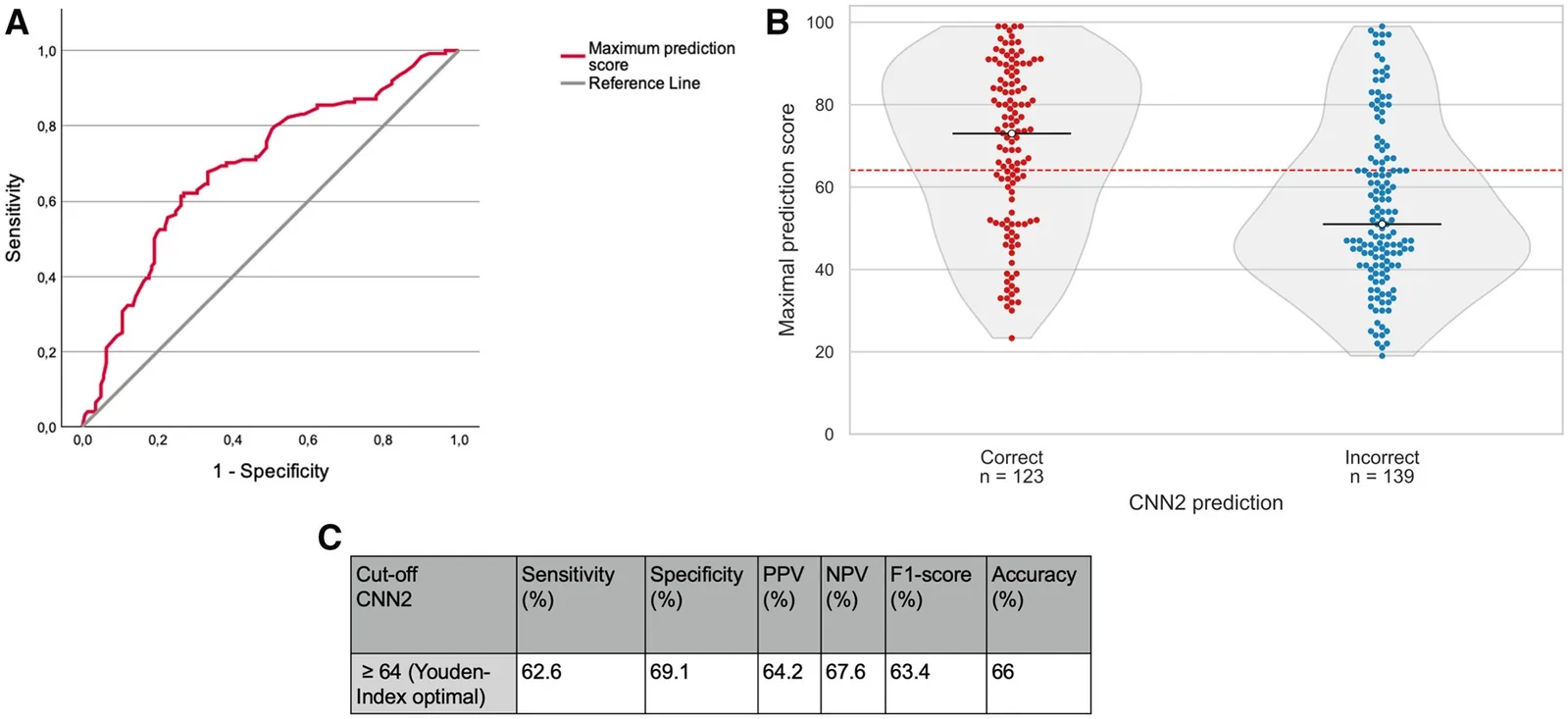
Performance and prediction confidence of CNN2 for histotype classification. (A) Receiver operating characteristic (ROC) analysis of prediction confidence. ROC curve assessing the ability of the maximal CNN2 prediction score to distinguish correct from incorrect histotype classifications (AUC = 0.73). (B) Distribution of maximal prediction scores. Violin plot showing the distribution of maximal prediction scores for correctly (*n* = 123) and incorrectly (*n* = 139) classified samples. The dashed line represents the optimal classification threshold (≥64) as determined by the Youden index. Horizontal bars indicate median. (C) Performance of prediction confidence at the Youden-index-derived threshold. At a maximal prediction-score cutoff of ≥64, sensitivity for identifying correct histotype predictions was 62.6%, specificity 69.1%, and overall accuracy 66.0%. The positive and negative predictive values were 64.2% and 67.6%, respectively, with an *F*1-score of 63.4%. These metrics describe the ability of prediction confidence to distinguish correct from incorrect entity assignments and do not represent binary tumor/non-tumor classification performance.

With regard to specific diagnostic performance of CNN2 in recurrent malignant gliomas in subgroup analysis, ROC analysis demonstrated excellent discriminative performance with an AUC of 0.899 (95% CI, 0.854-0.944) (Figure S1A). 48/71 images (67.6%) were correctly identified, with gliosis representing the most frequent misclassification (8.5%). This corresponded to a sensitivity of 67.6%, specificity of 94.2%, PPV of 81.4%, NPV of 88.7%, an *F*1-score of 73.8%, and an overall accuracy of 87.0% (Figure S1D). Further subgroup analyses in meningioma and metastases are shown in Figure S1B-D.

### SRH Histomorphology of Recurrent Tumor and Radiation Necrosis

On histomorphological analysis in depth, SRH images revealed distinct but overlapping histopathological features of recurrent tumor and radiation necrosis (Figure 5). Tumor samples showed high cellular density, nuclear hyperchromasia and pleomorphism, and pseudopalisading necrosis, consistent with established features of recurrent tumors.^27^^,^^28^ Correctly identified radiation necrosis was characterized by homogeneous necrosis, paucicellularity, hemosiderin deposits, and vascular hyalinization, in line with known histopathological hallmarks of post-radiation injury.^29^^,^^30^ In contrast, misclassified necrosis contained necrotic areas interspersed with cell-rich foci mimicking tumor-like architecture. Furthermore, the histomorphological analysis underscored the significance of the sampling procedure and location to ensure diagnostic accuracy and quality control (Figure S2).

**Figure 5.**
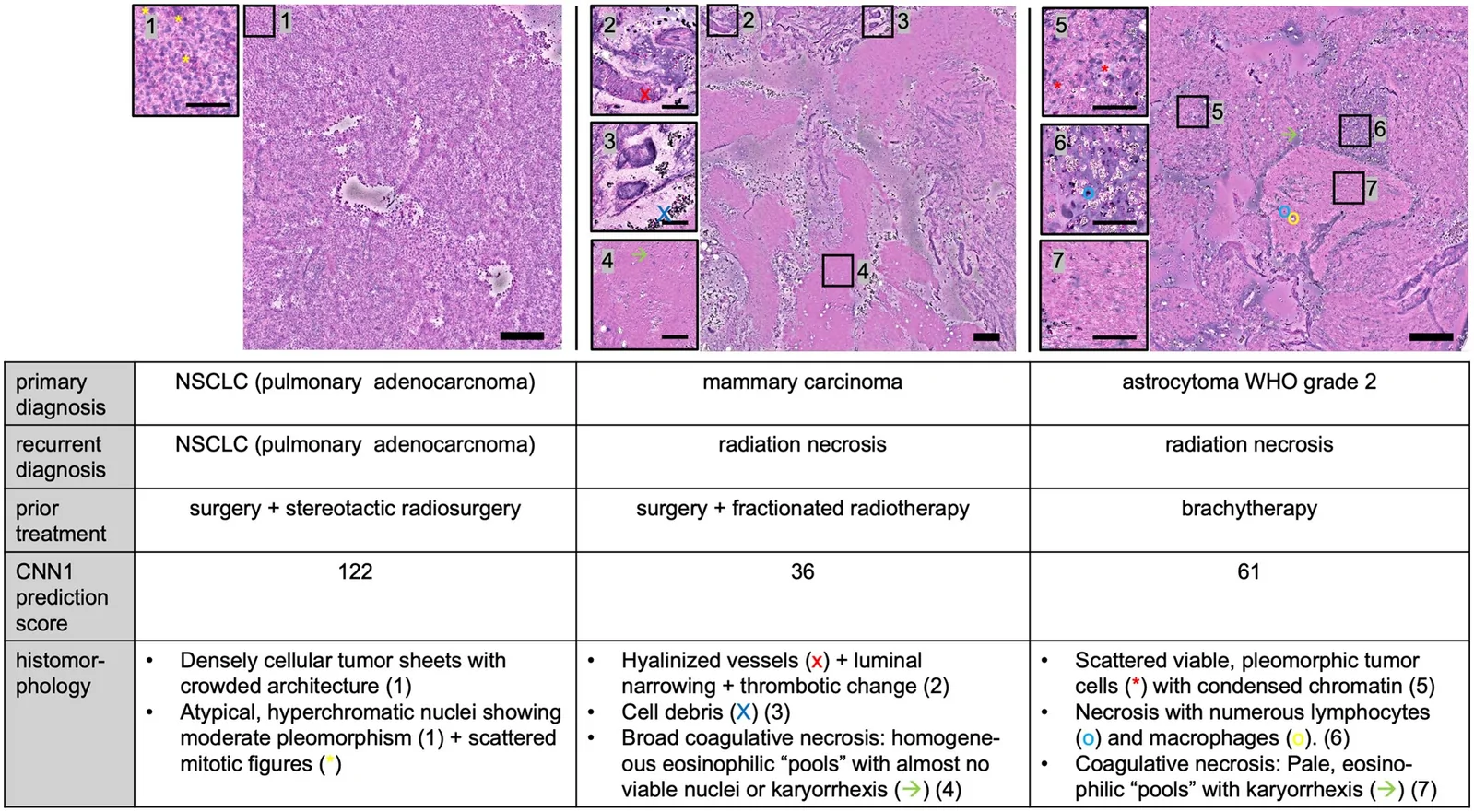
Challenging morphological overlaps of tumor and radiation necrosis. Representative SRH images illustrate the morphological overlap that challenges automated classification. The left panel shows an NSCLC recurrence (tumor probability score: 122) with high cellular density, atypical nuclei, and palisading necrosis, consistent with viable tumor. The middle panel depicts radiation necrosis correctly classified as non-tumor (mammary carcinoma, post-radiotherapy; score: 36) characterized by homogeneous necrosis, paucicellularity, and hemosiderin deposits. The right panel shows radiation necrosis (astrocytoma grade 2, brachytherapy; score: 61) misclassified as tumor, displaying necrotic regions interspersed with cell-rich foci that mimic hypercellular tumor areas. For figure preparation, SRH images were cropped and enlarged for visualization only; no sharpening, unsharp-mask filtering, or other image-enhancement procedures were applied. Large image scale bars = 200 µm; inset image scale bar = 20 µm.

### Influence of Pretreatment Effects on CNN Diagnostic Performance

We analyzed 212 SRH images allocated to three mutually exclusive previous treatment groups: surgery only (*n* = 34, 16.0%), surgery and radiotherapy (*n* = 73, 34.4%), and surgery, radiotherapy combined with medical treatment (*n* = 105, 49.5%).

For CNN1, accuracy was uniformly high and did not differ significantly across treatment groups: 91.2% (31/34) in the surgery-only group, 95.9% (70/73) in the prior bimodal treatment group, and 85.7% (90/105) in the prior trimodal treatment group (χ^2^ = 5.05, *P* = .080; linear trend χ^2^ = 2.39, *P* = .122) (Figure 2B). After adjustment for age and underlying pathology, no significant association between treatment group and model accuracy was observed. Model convergence was occasionally limited by the very high baseline performance, but all estimates consistently supported the absence of a therapy-related decline in tumor/non-tumor classification. Model calibration was excellent (Hosmer-Lemeshow *P* = .911), indicating no systematic deviation between observed and predicted probabilities, while the overall explanatory power remained low (Nagelkerke *R*^2^ = 0.058), reflecting the uniformly high baseline accuracy (overall 90.1%).

For CNN2, prediction accuracy declined with increasing multimodality: 61.8% (21/34) for surgery only, 50.7% (37/73) in the group of previous surgery and radiotherapy, and 39.0% (41/105) within the group of previous trimodal therapy (Figure 2B). The overall association was significant (χ^2^ = 6.04, *P* = .049), with a significant linear trend across therapy intensity (linear-by-linear association χ^2^ = 6.01, *P* = .014). In multivariable logistic regression adjusting for patient age and pathology, prior multimodal therapy remained independently associated with lower odds of a correct CNN2 prediction: bimodal therapy (surgery and radiotherapy) vs. surgery only, adjusted OR = 0.10 (95% CI, 0.02-0.39; *P* = .001) and trimodal therapy (surgery, radiotherapy, and chemotherapy) vs. surgery only, adjusted OR = 0.04 (95% CI, 0.01-0.20; *P* < .001) (Figure 2C). Patient age showed a modest positive association with correct classification (OR per year = 1.04, 95% CI, 1.01-1.06; *P* = .011). Model calibration was adequate (Hosmer-Lemeshow *P* = .127), and the overall model fit was strong (Nagelkerke *R*^2^ = 0.519; overall accuracy = 78.3%), supporting a robust negative association between therapy intensity and CNN2 performance.

In logistic regression analyses adjusting for maximal probability score, center (Freiburg vs. Cologne) was not an independent predictor of correct classification for either CNN (CNN2: OR 0.73, *P* = .27; CNN1: OR 1.07, *P* = .88).

## Discussion

This real-world study demonstrates that broad tumor recognition by SRH-based CNNs remains robust in recurrent brain tumors, whereas fine-grained tumor entity classification is more vulnerable to treatment-related tissue alterations. CNN1 maintained high tumor/non-tumor performance across prior-treatment groups when diagnostic image quality was sufficient. Similarly, when CNN2 output classes were collapsed into tumor and non-tumor categories, most tumor samples were correctly assigned to a tumorous class. In contrast, correct histotype classification by CNN2 was substantially lower and declined stepwise with increasing treatment intensity, independent of patient age and tumor entity. These findings suggest that the principal limitation of CNN2 in recurrent tumors concerns reliable assignment of the correct tumor entity rather than broad recognition of tumorous tissue. This pattern differs from previously reported model performance in cohorts dominated by treatment-naïve tissue.^20^^,^^21^^,^^31^ One likely contributor is the limited representation of recurrent and treatment-altered tissue in the datasets used for training of the existing models, accounting for approximately 10% of cases in Reinecke et al^21^ and 5% in Hollon et al.^20^ As a result, these models were not specifically optimized for the morphological spectrum encountered in recurrent disease, including radiation necrosis, reactive gliosis, and mixed viable and treatment-altered tumor tissue. Such overlap may particularly impair fine-grained entity classification and likely contribute to the therapy-associated decline in CNN2 performance observed in the present cohort. Recurrent tumors are generally characterized by high cellularity, nuclear pleomorphism, mitotic activity, pseudopalisading necrosis, and microvascular proliferation, while radiation necrosis is defined by paucicellularity, amorphous eosinophilic necrosis, vascular hyalinization, hemosiderin deposits, and reactive gliosis with macrophage-rich infiltrates.^30^^,^^32^ Both diagnoses can present with reactive gliosis and vascular alterations that complicate reliable differentiation. In radiation necrosis, clusters of reactive astrocytes and macrophages may mimic the cellular density of neoplastic tissue, while vascular hyalinization and fibrinoid necrosis can resemble the microvascular proliferation typically seen in high-grade gliomas. Conversely, tumor-associated necrosis, particularly pseudopalisading necrosis in glioblastoma, can appear morphologically similar to amorphous eosinophilic necrotic tissue in radiation injury.^29^ These shared features may confound CNN algorithms, which tend to misclassify reactive or necrotic tissue as tumor, as observed in this study. These findings further support the development of a recurrence-specific SRH model explicitly trained to distinguish viable recurrent tumor, radiation necrosis, and other non-neoplastic tissue. Such a task-specific approach may better reflect the actual intraoperative diagnostic challenge in recurrent brain tumor surgery than recalibration of an existing binary tumor/non-tumor classifier alone.

The strong influence of prior therapy on the performance of the CNN2 model observed here suggests that diagnostic entity recognition is particularly fragile in the presence of therapy-related alterations, reflecting the increased morphological complexity induced by radiation and medical therapy. Although the primary purpose of CNN2 is histotype classification, its output may also provide complementary information on tumor presence by indicating whether a tumorous or non-tumorous class is predicted. In the recurrent setting, however, the clinically relevant question for gliomas and meningiomas may extend beyond broad entity recognition to the detection of malignant progression, which is not represented in the current CNN2 taxonomy and requires task-specific model adaptation. Future model development should therefore emphasize both balanced inclusion of recurrent and treatment-altered samples and task-specific adaptation to clinically relevant questions in recurrent disease. Moreover, paired primary and recurrent samples from the same tumors were not available, precluding a direct assessment of whether CNN2 could capture within-patient tumor evolution or malignant progression over time.

Several limitations of the study need to be acknowledged. One limitation concerns the reference standard itself in recurrent disease. Therapy-induced tissue alterations, including fibrosis, microcalcification, neovascularization, and necrosis, often intermingle with viable tumor, creating “noisy” labels that challenge neuropathological interpretation and further serving as ground truth for AI model training.^17^

Intratumoral heterogeneity (see also Figure S2) represents another confounder, inducing sampling errors and bias. Differences between curated primary datasets and real-world application may be particularly relevant, as the latter exposes the algorithms to the full morphological spectrum of individual lesions rather than diagnostically selected image regions. This may contribute to underperformance in samples with spatially intermixed recurrent tumor, treatment-related alterations, and necrosis and may introduce mislabeling and sampling bias. To address this confounder, all SRH images underwent blinded consensus review by two neuropathologists, and tumor-free samples were deliberately included as negative controls. This procedure allowed us to test whether the CNN correctly refrained from classifying non-neoplastic tissue as tumor and to characterize false-positive predictions in the intraoperative setting. Nevertheless, future studies are needed to incorporate explicit annotation of mixed samples to further mitigate this potential bias. Furthermore, the limited tissue area interrogated by each SRH acquisition may not capture the full spatial heterogeneity of recurrent tumors, making representative intraoperative tissue sampling an important additional determinant of diagnostic performance.

Another limitation is class imbalance. Previous AI-assisted SRH studies have focused on the most common brain tumor entities, leaving rarer variants underrepresented. This imbalance carries the risk of systematic misclassification of rare tumors, as already observed in large SRH datasets with high overall accuracy but variable performance across smaller subgroups.^20^ Strategies such as oversampling rare classes, continuous expansion of the taxonomy, and explicit out-of-distribution detection, are essential to address this issue in the future.

In our recurrent tumor cohort, an exploratory cohort-derived tumor probability threshold of ≥59 for CNN1 yielded higher specificity than the conventional threshold of 40 used in primary glioma surgery, while maintaining high sensitivity. However, as this threshold was derived and evaluated within the same cohort, independent validation in a separate recurrence cohort is required. These findings underscore that probability thresholds optimized in treatment-naïve primary tumors may not be directly transferable to the biologically and histologically altered recurrence setting. In recurrent tumors, prior therapy frequently results in an overall reduction in viable tumor cellularity with pronounced spatial heterogeneity, characterized by focal residual tumor intermixed with necrosis, gliosis, and inflammatory tissue.^29^^,^^32^ This diminished and heterogeneous cellular signal likely shifts CNN output probabilities toward intermediate values, thereby necessitating a higher decision threshold to reliably distinguish viable tumor from non-neoplastic treatment-related changes compared with the lower cutoff commonly applied in primary glioma surgery.

In the meningioma subgroup, the predominance of recurrent disease likely introduced a systematic bias, as these specimens were statistically enriched for CNS WHO 2 and 3 tumors, susceptible in particular to tumor recurrence. Higher-grade meningiomas are characterized by increased cellular atypia, higher mitotic activity, and greater histopathological variability compared to more benign CNS WHO grade 1 lesions.^33^ This relative overrepresentation of atypical and anaplastic meningiomas may explain the diagnostic performance in this subgroup found to be lower than expected, compared to Hollon et al^20^ since the CNN2 was trained on predominating rather benign CNS WHO grade 1 meningiomas.

Domain shift is another important factor. Even though SRH provides label-free images, technical variations between laser systems, optical setups, and tissue handling may influence image characteristics, potentially impairing model transferability across centers.^34^^,^^35^ In the present study, however, no center-dependent differences in diagnostic performance were observed, suggesting robustness of both CNNs across acquisition sites despite differences in local workflows and case composition. Multicenter validation, standardized protocols, and automated quality control pipelines are needed to guarantee robustness in clinical deployment.

Image quality emerged as a major limitation of AI-assisted SRH diagnostics in the recurrent tumor setting, with nearly one-third of acquired images considered insufficient for diagnostic interpretation. Importantly, we found that the minimum number of high-quality patches required by the existing workflow did not provide sufficient image-level quality control in this recurrent tumor cohort. A proportion-based image-quality metric may be better suited to account for variation in SRH image size than an absolute minimum-patch prerequisite, as it relates the number of low-quality patches to the total number of analyzed patches. If the Youden-index-derived threshold of a maximum of 21.6% low-quality patches were applied as an image-level quality gate, the majority of low-quality images would have been identified and excluded from downstream AI-based tissue diagnostics. Establishing a stringent image-level quality-control strategy within the diagnostic SRH workflow for recurrent brain tumors could trigger repeat sampling and additional image acquisition in such cases, thereby helping to ensure that intraoperative decision-making is based on diagnostically sufficient images.

These findings are important when interpreting the diagnostic performance of CNNs. The reported classification accuracies are conditional on sufficient SRH image quality and should not be interpreted as the end-to-end accuracy of a fully automated acquisition-to-diagnosis workflow. Diagnostic correctness could not be validly assessed for images falsely accepted by the automated quality criterion because these images lacked a reliable image-level reference diagnosis, and systematic repeat SRH acquisition was not performed. Future prospective quality-control strategies should prioritize minimizing false acceptance of diagnostically insufficient images, even at the expense of increased repeat image acquisition, and require independent validation before clinical deployment.

## Conclusion

AI-assisted SRH provides robust broad CNS tumor recognition in recurrent disease when diagnostic image quality is sufficient, but current limitations remain in fine-grained entity classification and automated image-quality control. Prospective validation and adaptation of both diagnostic models and more stringent quality-control strategies to treatment-altered tissue will be essential for reliable clinical implementation.

## Supplementary Material

vdag267_Supplementary_Data

## Data Availability

The deidentified data supporting the findings of this study are available from the corresponding author upon reasonable request, subject to institutional, ethical, and data-protection requirements. The data are not publicly available because they contain patient-level clinical information.
