# Supplementary material for: Performance of intraoperative artificial intelligence-assisted label-free stimulated Raman histology in recurrent brain tumors
Source: Neurooncol Adv. 2026 Oct 29;8(1):vdag267. doi: 10.1093/noajnl/vdag267 (PMC13739048; doi:10.1093/noajnl/vdag267)
Supplement: Supplementary file 1 [file vdag267_supplementary_data.zip › Supplementary_Table_2_NOA-D-26-00448R1.docx]

Supplementary Table 2. Histopathological distribution of patients and SRH images in the Freiburg cohort.

| **Entity** | **Patients, n** | **Patients, %** | **SRH images, n** | **SRH images, %** |
| --- | --- | --- | --- | --- |
| **Recurrent tumors** | | | | |
| **Malignant glioma** | | | | |
| Oligodendroglioma CNS WHO 3 | 2 | 3.57 | 2 | 2.25 |
| Glioblastoma CNS WHO 4 | 16 | 28.57 | 27 | 30.34 |
| Gliosarcoma CNS WHO 4 | 1 | 1.79 | 1 | 1.12 |
| High-grade astrocytoma with piloid features | 1 | 1.79 | 1 | 1.12 |
| Astroblastoma | 1 | 1.79 | 2 | 2.25 |
| **Low-grade glioma** | | | | |
| Oligodendroglioma CNS WHO 2 | 1 | 1.79 | 1 | 1.12 |
| **Metastases** | | | | |
| NSCLC | 3 | 5.36 | 6 | 6.74 |
| SCLC | 1 | 1.79 | 3 | 3.37 |
| Prostate carcinoma | 1 | 1.79 | 1 | 1.12 |
| **Meningioma** | | | | |
| CNS WHO 1 | 2 | 3.57 | 3 | 3.37 |
| CNS WHO 2 | 5 | 8.93 | 7 | 7.87 |
| CNS WHO 3 | 4 | 7.14 | 7 | 7.87 |
| **Other glial tumors** | | | | |
| Ependymoma CNS WHO 3 | 1 | 1.79 | 1 | 1.12 |
| **Pituitary tumors** | | | | |
| Pituitary neuroendocrine tumors | 3 | 5.36 | 5 | 5.62 |
| **No tumor** | | | | |
| Radiation necrosis | 3 | 5.36 | 4 | 4.49 |
| Gliosis/no tumor | 11 | 19.64 | 18 | 20.22 |
| **Total** | **56** | **100.00** | **89** | **100.00** |
