# Supplementary material for: Performance of intraoperative artificial intelligence-assisted label-free stimulated Raman histology in recurrent brain tumors
Source: Neurooncol Adv. 2026 Oct 29;8(1):vdag267. doi: 10.1093/noajnl/vdag267 (PMC13739048; doi:10.1093/noajnl/vdag267)
Supplement: Supplementary file 1 [file vdag267_supplementary_data.zip › Supplemental_Results_NOA-D-26-00448R1_CLEAR.docx]

**Supplemental Results**

In binary prediction analysis (tumorous vs. non-tumorous prediction classes) the CNN2 correctly classified 57/75 tumor-free samples (76%) as gray or white matter or gliosis, but only half of the images of radiation necrosis (7/14) were correctly identified as non-tumor tissue. The vast majority of 178/187 (95.2%) tumor samples were correctly assigned to one of the tumorous prediction classes disregarding the correct entity itself among those. Using the CNN2 merely as screening method for such binary prediction (tumor vs. non-tumorous prediction classes) the sensitivity was 95.2%, specificity 76% with a positive predictive value for tumor of 90.8% and a negative predictive value of 86.4%. The F1-score was 93% and overall accuracy was 89.7%.

**Supplemental figure 1**

**ROC analyses and performance metrics of CNN2 for entity-specific classification.**

(A) ROC curve for malignant glioma prediction (AUC = 0.899, 95% CI 0.855–0.944).

(B) ROC curve for brain metastasis prediction (AUC = 0.869, 95% CI 0.805–0.932).

(C) ROC curve for meningioma prediction (AUC = 0.956, 95% CI 0.931–0.981).

(D) Performance table summarizing AUC values, sensitivity, specificity, PPV, NPV, F1-score, and accuracy for each diagnostic subgroup.

**Alt text:** Receiver operating characteristic curves and performance metrics for entity-specific CNN classification of recurrent malignant glioma, brain metastasis, and meningioma. Discriminative performance differs across tumor entities, with the highest area under the curve observed for meningioma.

**Supplemental figure 2**

**Challenging intratumoral heterogeneity**

Two adjacent regions from the same specimen demonstrate marked histological heterogeneity. The left panel shows radiation necrosis (tumor probability score: 47) with disorganized, cell-poor necrotic tissue lacking viable tumor nests, consistent with post-radiation injury and leading to misclassification. The right panel depicts viable tumor (score: 59) with high cellular density, nuclear pleomorphism, hyperchromasia, and pseudopalisading necrosis. This juxtaposition highlights intratumoral heterogeneity as a major confounder for both histopathological evaluation and AI-based classification. For figure preparation, SRH images were cropped and enlarged for visualization only; no sharpening, unsharp-mask filtering, or other image-enhancement procedures were applied.

Large image scale bars = 200 µm; inset image scale bar = 20 µm.

**Alt text:** Two adjacent stimulated Raman histology regions from the same specimen demonstrate marked spatial heterogeneity. One region contains predominantly cell-poor radiation necrosis, whereas the other shows viable hypercellular tumor with nuclear atypia and pseudopalisading necrosis.

**Supplementary Table 1.**

**Histopathological distribution of patients and SRH images in the Cologne cohort.**
Number of patients and corresponding SRH images stratified by final histopathological diagnosis for the University Hospital Cologne cohort.

**Supplementary Table 2.**

**Histopathological distribution of patients and SRH images in the Freiburg cohort.**
Number of patients and corresponding SRH images stratified by final histopathological diagnosis for the University Hospital Freiburg cohort.
