# Supplementary material for: Performance of intraoperative artificial intelligence-assisted label-free stimulated Raman histology in recurrent brain tumors
Source: Neurooncol Adv. 2026 Oct 29;8(1):vdag267. doi: 10.1093/noajnl/vdag267 (PMC13739048; doi:10.1093/noajnl/vdag267)
Supplement: Supplementary file 1 [file vdag267_supplementary_data.zip › Supplementary_Table_1_NOA-D-26-00448R1.docx]

Supplementary Table 1. Histopathological distribution of patients and SRH images in the Cologne cohort.

| **Entity** | **Patients, n** | **Patients, %** | **SRH images, n** | **SRH images, %** |
| --- | --- | --- | --- | --- |
| **Recurrent tumors** | | | | |
| **Malignant glioma** | | | | |
| Oligodendroglioma CNS WHO 3 | 3 | 3.45 | 6 | 3.47 |
| Astrocytoma CNS WHO 3 | 4 | 4.60 | 5 | 2.89 |
| Astrocytoma CNS WHO 4 | 3 | 3.45 | 3 | 1.73 |
| Glioblastoma CNS WHO 4 | 11 | 12.64 | 22 | 12.72 |
| Pleomorphic xanthoastrocytoma CNS WHO 3 | 1 | 1.15 | 1 | 0.58 |
| Pediatric-type diffuse high-grade glioma | 1 | 1.15 | 1 | 0.58 |
| **Low-grade glioma** | | | | |
| Oligodendroglioma CNS WHO 2 | 2 | 2.30 | 5 | 2.89 |
| Astrocytoma CNS WHO 2 | 2 | 2.30 | 2 | 1.16 |
| **Metastases** | | | | |
| NSCLC | 8 | 9.20 | 16 | 9.25 |
| SCLC | 3 | 3.45 | 6 | 3.47 |
| Melanoma | 2 | 2.30 | 5 | 2.89 |
| Mammary carcinoma | 2 | 2.30 | 2 | 1.16 |
| Adenocarcinoma of the gastrointestinal tract | 2 | 2.30 | 5 | 2.89 |
| Large-cell NET | 1 | 1.15 | 3 | 1.73 |
| **Meningioma** | | | | |
| CNS WHO 1 | 5 | 5.75 | 9 | 5.20 |
| CNS WHO 2 | 11 | 12.64 | 22 | 12.72 |
| CNS WHO 3 | 2 | 2.30 | 5 | 2.89 |
| **Other glial tumors** | | | | |
| Ependymoma CNS WHO 3 | 1 | 1.15 | 1 | 0.58 |
| **Pituitary tumors** | | | | |
| Pituitary neuroendocrine tumors | 1 | 1.15 | 1 | 0.58 |
| **No tumor** | | | | |
| Radiation necrosis | 6 | 6.90 | 10 | 5.78 |
| Gliosis/no tumor | 16 | 18.39 | 43 | 24.86 |
| **Total** | **87** | **100.00** | **173** | **100.00** |
